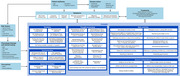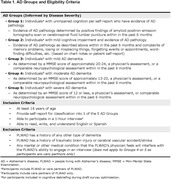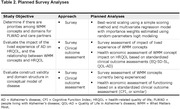# Measuring What Matters Most to People Living with Alzheimer’s Disease and Care Partners: What Matters Most Quantitative Research Development

**DOI:** 10.1002/alz.085103

**Published:** 2025-01-09

**Authors:** Carla (DeMuro) Romano, Emily Bratlee‐Whitaker, Ann Hartry, Elizabeth S Mearns, Jim Taylor, Geri Taylor, Leigh F Callahan, Doreen Monks, Ian Kremer, Debra Lappin, Theresa Frangiosa, Sanjyot Sangodkar, William L Herring, Kajan Gnanasakthy, Diana Goss, Lori McLeod, Teresa Edwards, Christie Poulos, Dana DiBenedetti, Russ Paulsen

**Affiliations:** ^1^ RTI Health Solutions, Research Triangle Park, NC USA; ^2^ Biogen Inc, Cambridge, MA USA; ^3^ Genentech, Inc., South San Francisco, CA USA; ^4^ Memory Advocate Peers (MAP), New York, NY USA; ^5^ University of North Carolina, Chapel Hill, NC USA; ^6^ Advocate and person living with Alzheimer’s disease, Livingston, NJ USA; ^7^ LEAD Coalition (Leaders Engaged on Alzheimer’s Disease), Washington, DC USA; ^8^ Lappin Kramer LLC, Denver, CO USA; ^9^ Faegre Drinker Consulting, Washington, DC USA; ^10^ Karolinska Institute, Stockholm, Södermanland and Uppland Sweden; ^11^ RTI Health Solutoins, Research Triangle Park, NC USA; ^12^ UsAgainstAlzheimer’s, Washington, DC USA

## Abstract

**Background:**

Previous What Matters Most (WMM) research identified and verified patient‐important concepts among a diverse population of people living with Alzheimer’s disease (PLWAD) and their care partners across all stages of Alzheimer’s disease (AD). Additionally, a conceptual model of disease was developed using hypothesized domains and was further refined using qualitative data (Figure 1). This next phase of WMM research aims to quantify the importance of concepts, impacts, and outcomes and to determine priorities among PLWAD and care partners within a diverse sample including underrepresented ethnic and racial groups at each level of disease severity.

**Methods:**

A cross‐sectional, observational, web‐based survey will be administered to up to 600 adults ≥18 years living with or at risk for AD and care partners of individuals across the disease spectrum. We aim to sample an equal distribution of participants categorized in 1 of 5 AD groups (Table 1) maximizing representation of educational levels, socioeconomic status, and ages, and to include approximately 50% participants who self‐identify as Black, Asian, Hispanic, or a mixed ethnicity. The survey builds upon an initial rating study (Hauber et al., 2023. *Neurol Ther*;12:505‐527) to (1) determine ranked priorities among WMM concepts, (2) evaluate the impact of the lived experience of concepts, and (3) evaluate construct validity of specific hypothesized domains, alongside clinical outcome assessments to facilitate health economic assessment (Table 2). A draft survey will be refined following cognitive debriefing with small samples across groups 1‐5 before completion by our target sample. Data may be stratified by AD group or background factors (e.g., ethnicity, education) for subgroup analyses, sample sizes permitting.

**Results:**

Anticipated survey and clinical outcome assessment findings will provide rankings of concepts, impacts, and outcomes among a diverse and inclusive population of PLWAD and care partners across the spectrum of disease.

**Conclusions:**

This research expands upon prior identification and verification of WMM concepts to determine priorities and characterize the lived experience of PLWAD and care partners across diverse and inclusive populations. Data are intended to inform treatment‐related needs, selection of important patient‐centric outcome measures, and study endpoints to better guide the development of AD treatments and services.